# Supplementary material for: Systematic elucidation of independently modulated genes in Lactiplantibacillus plantarum reveals a trade‐off between secondary and primary metabolism
Source: Microb Biotechnol. 2024 Feb 23;17(2):e14425. doi: 10.1111/1751-7915.14425 (PMC10886434; doi:10.1111/1751-7915.14425)
Supplement: Supplementary file 1 — Data S1.. [file MBT2-17-e14425-s001.docx]

Systematic elucidation of independently modulated genes in *Lactiplantibacillus plantarum* reveals a trade-off between secondary and primary metabolisms

## Authorship

Sizhe Qiu^1,2^, Yidi Huang^3^, Shishun Liang^4^, Hong Zeng^2^*, Aidong Yang^1^*

^1^Department of Engineering Science, University of Oxford, OX1 3PJ, United Kingdom

^2^School of Food and Health, Beijing Technology and Business University, Beijing 100048, China

^3^School of Computer Science and Engineering, Beijing 100191, Beihang University

^4^ Department of Life Science, Imperial College London, SW7 2AZ, United Kingdom

*Corresponding author: [zenghong@btbu.edu.cn](mailto:zenghong@btbu.edu.cn) (H. Zeng); [aidong.yang@eng.ox.ac.uk](mailto:aidong.yang@eng.ox.ac.uk) (A. Yang)

##

## 1. Tables

| Table S1. Metadata of sample conditions | | | |
| --- | --- | --- | --- |
| Sample | Condition | Cultivation time | Reference |
| wt_pH6.2 | MRS medium, 10 μg/L valinomycin, pH adjusted to 6.2 with addition of lactic acid | 12 hours | [(Jung & Lee, 2020)](https://paperpile.com/c/MkYOqv/1N2V) |
| wt_pH5.5 | MRS medium, 10 μg/L valinomycin, pH adjusted to 5.5 with addition of lactic acid | 12 hours | [(Jung & Lee, 2020)](https://paperpile.com/c/MkYOqv/1N2V) |
| wt_pH5.0 | MRS medium, 10 μg/L valinomycin, pH adjusted to 5.0 with addition of lactic acid | 12 hours | [(Jung & Lee, 2020)](https://paperpile.com/c/MkYOqv/1N2V) |
| wt_3OCp1 | MRS medium, 100 μM 3OC12, 37℃, +1 hour | _ | [(Spangler et al., 2019)](https://paperpile.com/c/MkYOqv/btSD) |
| wt_3OCp4 | MRS medium, 100 μM 3OC12, 37℃, +4 hour | _ | [(Spangler et al., 2019)](https://paperpile.com/c/MkYOqv/btSD) |
| wt_3OCp7 | MRS medium, 100 μM 3OC12, 37℃, +7 hour | _ | [(Spangler et al., 2019)](https://paperpile.com/c/MkYOqv/btSD) |
| wt_3OCm1 | MRS medium, 0.1% DMSO (v/v), 37℃, +7 hour | _ | [(Spangler et al., 2019)](https://paperpile.com/c/MkYOqv/btSD) |
| wt_3OCm4 | MRS medium, 0.1% DMSO (v/v), 37℃, +7 hour | _ | [(Spangler et al., 2019)](https://paperpile.com/c/MkYOqv/btSD) |
| wt_3OCm7 | MRS medium, 0.1% DMSO (v/v), 37℃, +7 hour | _ | [(Spangler et al., 2019)](https://paperpile.com/c/MkYOqv/btSD) |
| WCFS1_MRS | MRS broth, pH = 5.7, 30℃ | 24 hours | [(Filannino et al., 2018)](https://paperpile.com/c/MkYOqv/c89u) |
| LB16_MRS | MRS broth, pH = 5.7, 30℃ | 24 hours | [(Filannino et al., 2018)](https://paperpile.com/c/MkYOqv/c89u) |
| BEE | Homogenized bee:water of 3:1 (v/v), pH = 4.7, 30℃ | 24 hours | [(Filannino et al., 2018)](https://paperpile.com/c/MkYOqv/c89u) |
| CB | Cheese broth prepared as in Neviani *et al.*, 2009 [(Neviani et al., 2009)](https://paperpile.com/c/MkYOqv/H9gF), pH = 6.1, 30℃ | 24 hours | [(Filannino et al., 2018)](https://paperpile.com/c/MkYOqv/c89u) |
| DE | Homogenized larvae:water of 1:1 (wt/wt), pH = 6.5, 30℃ | 24 hours | [(Filannino et al., 2018)](https://paperpile.com/c/MkYOqv/c89u) |
| FE | Fecal extract, pH = 5.9, 30℃ | 24 hours | [(Filannino et al., 2018)](https://paperpile.com/c/MkYOqv/c89u) |
| OE | Homogenized olive extract, pH = 4.7, 30℃ | 24 hours | [(Filannino et al., 2018)](https://paperpile.com/c/MkYOqv/c89u) |
| PJ | Pineapple juice medium treated as in Filannino *et al.*, 2014 [(Filannino et al., 2014)](https://paperpile.com/c/MkYOqv/OHL7), pH = 3.5, 30℃ | 24 hours | [(Filannino et al., 2018)](https://paperpile.com/c/MkYOqv/c89u) |
| TJ | Tomato juice medium treated as in Filannino *et al.*, 2014 [(Filannino et al., 2014)](https://paperpile.com/c/MkYOqv/OHL7), pH = 4.7, 30℃ | 24 hours | [(Filannino et al., 2018)](https://paperpile.com/c/MkYOqv/c89u) |
| WFH | Wheat flour hydrolyzate treated as in Gobbetti *et al.*, 1994 [(Gobbetti et al., 1994)](https://paperpile.com/c/MkYOqv/FC9R), pH = 5.6, 30℃ | 24 hours | [(Filannino et al., 2018)](https://paperpile.com/c/MkYOqv/c89u) |
| wt_GLC | MRS medium, 0.05% (w/v) L-cysteine HCl, 1% (w/v) glucose, 37℃ | 48 hours | [(Özcan et al., 2021)](https://paperpile.com/c/MkYOqv/QEJ2) |
| wt_HMO | MRS medium, 0.05% (w/v) L-cysteine HCl, 1% (w/v) human milk oligosaccharides, 37℃ | 48 hours | [(Özcan et al., 2021)](https://paperpile.com/c/MkYOqv/QEJ2) |
| wt_PAC1 | MRS medium, 0.05% (w/v) L-cysteine HCl, 1% (w/v) proanthocyanidin fraction 1, 37℃ | 48 hours | [(Özcan et al., 2021)](https://paperpile.com/c/MkYOqv/QEJ2) |
| wt_PAC2 | MRS medium, 0.05% (w/v) L-cysteine HCl, 1% (w/v) proanthocyanidin fraction 2, 37℃ | 48 hours | [(Özcan et al., 2021)](https://paperpile.com/c/MkYOqv/QEJ2) |
| wt_HMOPAC1 | MRS medium, 0.05% (w/v) L-cysteine HCl, 1% (w/v) human milk oligosaccharides + proanthocyanidin fraction 1, 37℃ | 48 hours | [(Özcan et al., 2021)](https://paperpile.com/c/MkYOqv/QEJ2) |
| wt_HMOPAC2 | MRS medium, 0.05% (w/v) L-cysteine HCl, 1% (w/v) human milk oligosaccharides + proanthocyanidin fraction 1, 37℃ | 48 hours | [(Özcan et al., 2021)](https://paperpile.com/c/MkYOqv/QEJ2) |
| wt_FOSPAC1 | MRS medium, 0.05% (w/v) L-cysteine HCl, 1% (w/v) fructooligosaccharides + proanthocyanidin fraction 1, 37℃ | 48 hours | [(Özcan et al., 2021)](https://paperpile.com/c/MkYOqv/QEJ2) |
| wt_FOSPAC2 | MRS medium, 0.05% (w/v) L-cysteine HCl, 1% (w/v) fructooligosaccharides + proanthocyanidin fraction 2, 37℃ | 48 hours | [(Özcan et al., 2021)](https://paperpile.com/c/MkYOqv/QEJ2) |
| wt_XG | MRS medium, 0.05% (w/v) L-cysteine HCl, 1% (w/v) xyloglucans, 37℃ | 48 hours | [(Özcan et al., 2021)](https://paperpile.com/c/MkYOqv/QEJ2) |
| wt_XGPAC2 | MRS medium, 0.05% (w/v) L-cysteine HCl, 1% (w/v) xyloglucans + proanthocyanidin fraction 1, 37℃ | 48 hours | [(Özcan et al., 2021)](https://paperpile.com/c/MkYOqv/QEJ2) |
| wt_XGPAC2 | MRS medium, 0.05% (w/v) L-cysteine HCl, 1% (w/v) xyloglucans + proanthocyanidin fraction 2, 37℃ | 48 hours | [(Özcan et al., 2021)](https://paperpile.com/c/MkYOqv/QEJ2) |

| Table S2. Metabolic reaction information | | | |
| --- | --- | --- | --- |
| ID | iModulon | Genes | Name |
| SERAT | McbR | lp_0254 | Serine O-acetyltransferase |
| CYSS | McbR | lp_0265 | Cysteine synthase |
| CYSTL | McbR | lp_0255 | Cystathionine-b-lyase |
| CYSTGL | McbR | lp_0255 | Cystathionine-b-lyase |
| ASPCT | PyrR | lp_2703 | Aspartate carbamoyltransferase |
| DHORTS | PyrR | lp_2702 | Dihydroorotase |
| DHORD6 | PyrR | lp_2699 | Dihydoorotic acid dehydrogenase |
| ORPT | _ | lp_2697 | Orotate phosphoribosyltransferase |
| OMPDC | PyrR | lp_2698 | Orotidine 5 phosphate decarboxylase |
| CBPS | PyrR, ArgR/MleR | lp_2700/2701, lp_0526/0527 | Carbamoyl-phosphate synthase (glutamine-hydrolysing) |
| ORNTAC | ArgR/MleR | lp_0529 | Ornithine transacetylase |
| ACOTA | ArgR/MleR | lp_0531 | Acetylornithine transaminase |
| ACGK | ArgR/MleR | lp_0530 | Acetylglutamate kinase |
| AGPR | ArgR/MleR | lp_0528 | N-acetyl-g-glutamyl-phosphate reductase |
| OCBT | ArgR/MleR | lp_0532 | Ornithine carbamoyltransferase |
| MALt2r | ArgR/MleR | lp_1119 | L-malate reversible transport via proton symport |
| MALLAC | ArgR/MleR | lp_1118 | Malolactic enzyme |
| PYROX | CcpA | lp_2629 | Pyruvate oxidase |
| PDH | CcpA | lp_2151-2154 | Pyruvate dehydrogenase |
| PFL | CcpA | lp_3313/3314 | Formate C acetyltransferase |
| GLYCt1 | CcpA | lp_0372 | Glycerol transport via channel |
| GLYK | CcpA | lp_0370 | Glycerol kinase |
| G3PO | CcpA | lp_0371 | Glycerol 3 phosphate oxidase |
| NDPK | CcpA | lp_0242 | Nucleoside diphosphate kinase |
| G1PTMT | GntR | lp_1186 | Glucose 1 phosphate thymidylyltransferase |
| TDPGDH | GntR | lp_1189 | DTDPglucose 4,6-dehydratase |
| TDPDRE | GntR | lp_1188 | DTDP-4-dehydrorhamnose 3,5-epimerase |
| TDPDRR | GntR | lp_1190 | DTDP-4-dehydrorhamnose reductase |
| CPSS | GntR | lp_1177-1185 | Capsular polysaccharide synthase complex |
| MANpts | GntR | lp_0576/0577 | D-mannose transport via PEP:Pyr PTS |
| ASP1DC | GntR | lp_0579 | Aspartate 1-decarboxylase |
| DB4PS | GntR | lp_1437 | 3,4-Dihydroxy-2-butanone-4-phosphate synthase |
| DHDPS | GntR | lp_2685 | Dihydrodipicolinate synthase |
| MELIBt2 | GalR/AraR | lp_3486 | Melibiose transport in via symport |
| RAFGH | GalR/AraR | lp_3485 | Raffinose galactohydrolase |
| GALS3 | GalR/AraR | lp_3485 | A-galactosidase (melibiose) |
| BGLA1 | GalR/AraR | lp_0440 | 6-phospho-beta-glucosidase |
| GALM | _ | lp_0826/1731/3487 | Aldose 1 epimerase |
| GALK2 | GalR/AraR | lp_3482 | Galactokinase |
| GALT | GalR/AraR | lp_3480 | Galactose 1 phosphate uridylyltransferase |
| UDPG4E | GalR/AraR | lp_3481 | UDPglucose 4 epimerase |
| SERt2r | GalR/AraR | lp_0502 | L-serine reversible transport via proton symport |
| SERD_L | GalR/AraR | lp_0505/0506 | L-serine deaminase |
| TKT1 | GalR/AraR | lp_3538 | Transketolase |
| TKT2 | GalR/AraR | lp_3538 | Transketolase |
| TALA | GalR/AraR | lp_3539 | Transaldolase |
| RBP4E | GalR/AraR | lp_3555 | L-ribulose-phosphate 4-epimerase |
| RBK_L1 | GalR/AraR | lp_3556 | L-ribulokinase (L-ribulose) |
| ARAI | GalR/AraR | lp_3554 | L-arabinose isomerase |
| ARAB_Lt | GalR/AraR | lp_3557 | L-arabinose extracellular transport |
| RBLK2 | GalR/AraR | lp_3556 | L-ribulokinase (ribitol) |

## 2. Figures


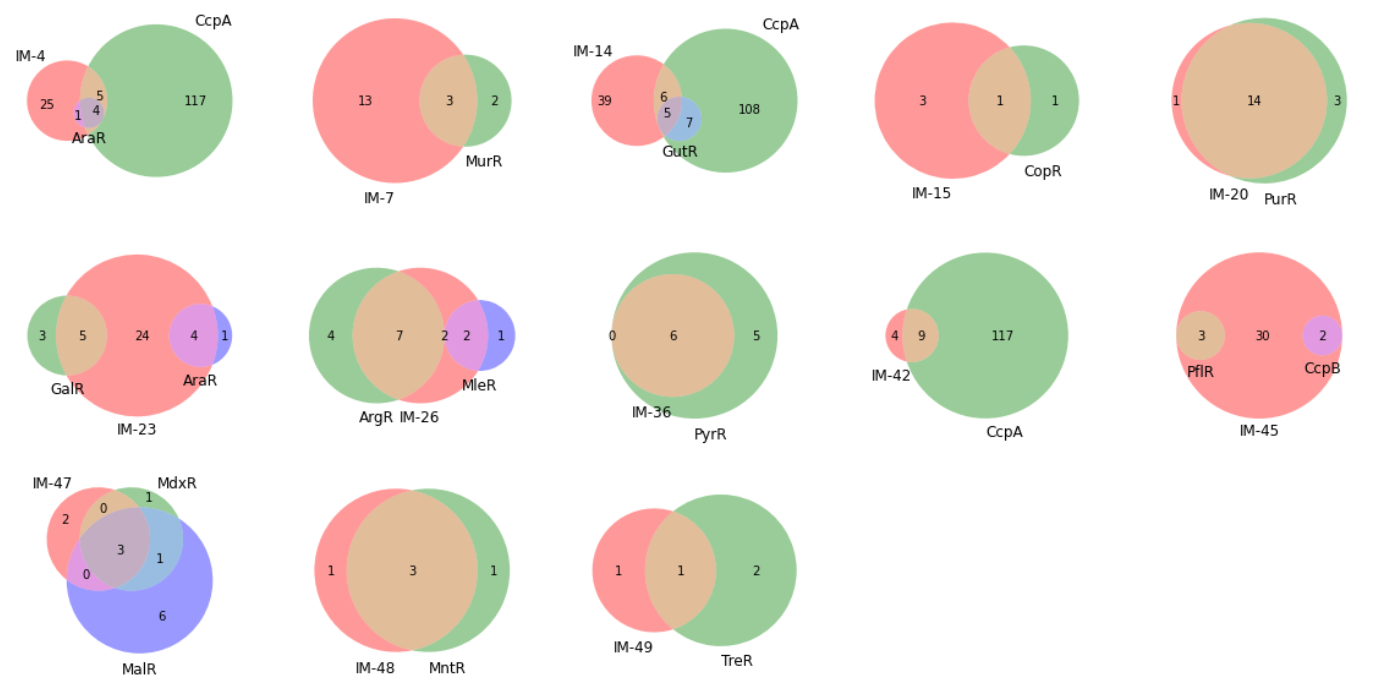


Figure S1. Venn diagrams of regulon enrichment for IM-4, 7, 14, 15, 20, 23, 26, 36, 42, 45, 47, 48, 49.


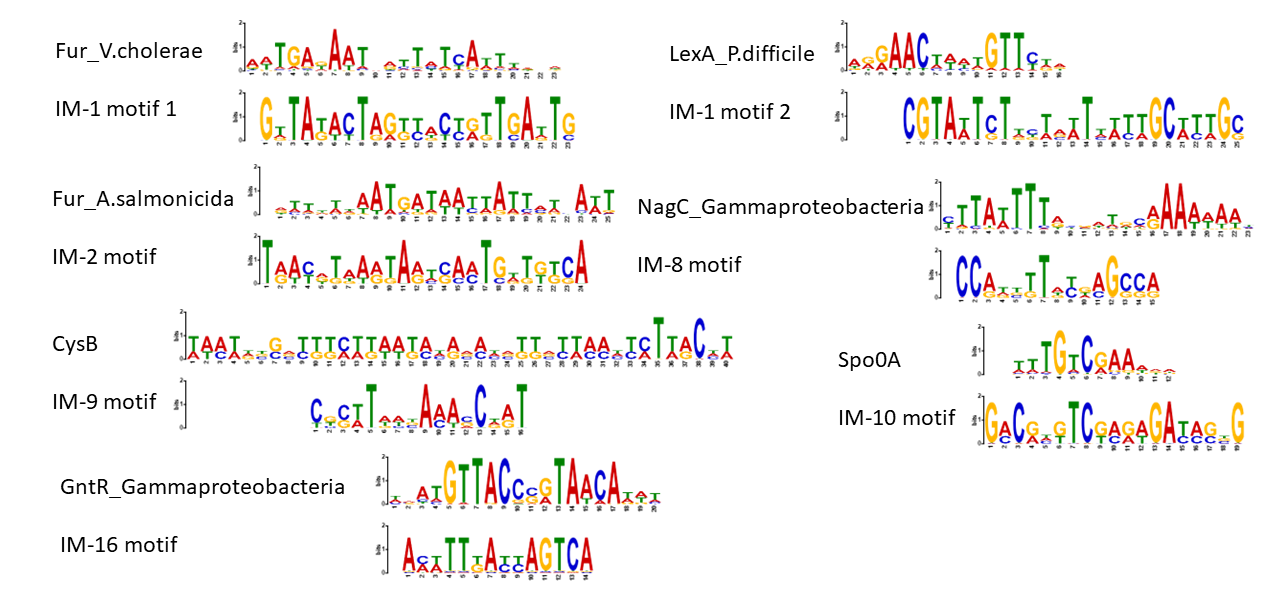


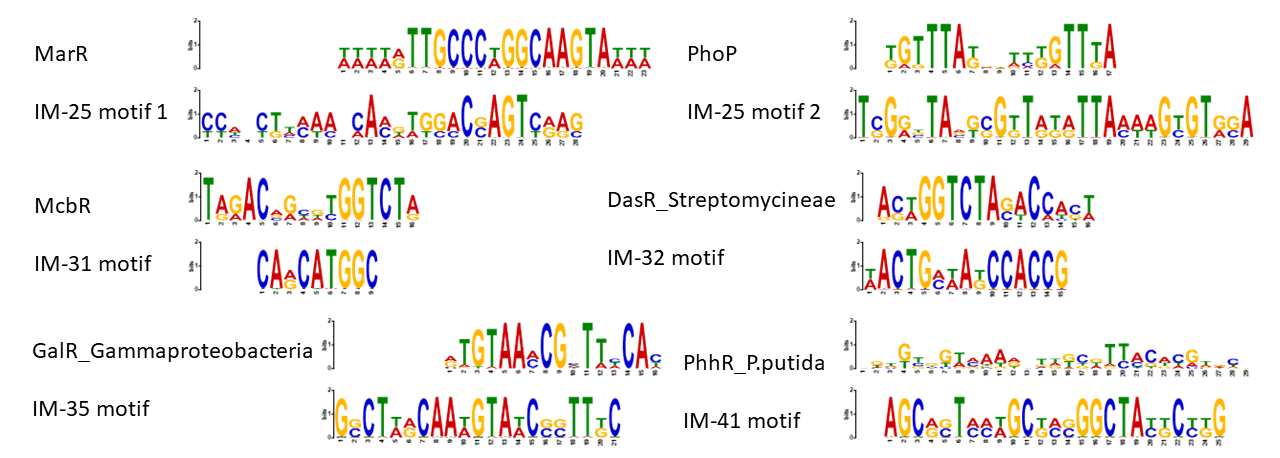


Figure S2. Motif comparison logo plots for IM-1, 2, 8, 9, 10, 16, 25, 31, 32, 35, 41.

## 3. Supplementary methods

### 3.1 Functional annotation of iModulons

Coding genes in the genome of Lactobacillus plantarum were annotated using eggNOG-mapper [(Cantalapiedra et al., 2021)](https://paperpile.com/c/MkYOqv/bTnoP), and COG (Clusters of Orthologous Groups), KEGG, CAZy and BiGG annotations were obtained. The E-value threshold was 1e-5. The thresholds of query and subject coverages were all set as 50%. The functional annotation of each iModulon was determined based on enrichment analysis of specific pathways.

### 3.2 Metabolic pathway visualization

Metabolic pathways encoded by iModudon member genes were visualized using Escher (<https://escher.github.io/>) [(King et al., 2015)](https://paperpile.com/c/MkYOqv/B0vE). The genome-scale metabolic model **iBT721** [(Teusink et al., 2006)](https://paperpile.com/c/MkYOqv/v8ba2) was used as the database of gene-reaction rules to query for metabolic reactions encoded by member genes of iModudons.

## References

[Cantalapiedra, C. P., Hernández-Plaza, A., Letunic, I., Bork, P., & Huerta-Cepas, J. (2021). eggNOG-mapper v2: Functional Annotation, Orthology Assignments, and Domain Prediction at the Metagenomic Scale. *Molecular Biology and Evolution*, *38*(12), 5825–5829.](http://paperpile.com/b/MkYOqv/bTnoP)

[Filannino, P., Cardinali, G., Rizzello, C. G., Buchin, S., De Angelis, M., Gobbetti, M., & Di Cagno, R. (2014). Metabolic responses of Lactobacillus plantarum strains during fermentation and storage of vegetable and fruit juices. *Applied and Environmental Microbiology*, *80*(7), 2206–2215.](http://paperpile.com/b/MkYOqv/OHL7)

[Filannino, P., De Angelis, M., Di Cagno, R., Gozzi, G., Riciputi, Y., & Gobbetti, M. (2018). How Lactobacillus plantarum shapes its transcriptome in response to contrasting habitats. *Environmental Microbiology*, *20*(10), 3700–3716.](http://paperpile.com/b/MkYOqv/c89u)

[Gobbetti, M., Corsetti, A., & Rossi, J. (1994). The sourdough microflora. Interactions between lactic acid bacteria and yeasts: metabolism of carbohydrates. *Applied Microbiology and Biotechnology*, *41*(4), 456–460.](http://paperpile.com/b/MkYOqv/FC9R)

[Jung, S., & Lee, J.-H. (2020). Characterization of transcriptional response of Lactobacillus plantarum under acidic conditions provides insight into bacterial adaptation in fermentative environments. *Scientific Reports*, *10*(1), 19203.](http://paperpile.com/b/MkYOqv/1N2V)

[King, Z. A., Dräger, A., Ebrahim, A., Sonnenschein, N., Lewis, N. E., & Palsson, B. O. (2015). Escher: A Web Application for Building, Sharing, and Embedding Data-Rich Visualizations of Biological Pathways. *PLoS Computational Biology*, *11*(8), e1004321.](http://paperpile.com/b/MkYOqv/B0vE)

[Neviani, E., De Dea Lindner, J., Bernini, V., & Gatti, M. (2009). Recovery and differentiation of long ripened cheese microflora through a new cheese-based cultural medium. *Food Microbiology*, *26*(3), 240–245.](http://paperpile.com/b/MkYOqv/H9gF)

[Özcan, E., Rozycki, M. R., & Sela, D. A. (2021). Cranberry Proanthocyanidins and Dietary Oligosaccharides Synergistically Modulate Lactobacillus plantarum Physiology. *Microorganisms*, *9*(3). https://doi.org/](http://paperpile.com/b/MkYOqv/QEJ2)[10.3390/microorganisms9030656](http://dx.doi.org/10.3390/microorganisms9030656)

[Spangler, J. R., Dean, S. N., Leary, D. H., & Walper, S. A. (2019). Response of Lactobacillus plantarum WCFS1 to the Gram-Negative Pathogen-Associated Quorum Sensing Molecule N-3-Oxododecanoyl Homoserine Lactone. *Frontiers in Microbiology*, *10*, 715.](http://paperpile.com/b/MkYOqv/btSD)

[Teusink, B., Wiersma, A., Molenaar, D., Francke, C., de Vos, W. M., Siezen, R. J., & Smid, E. J. (2006). Analysis of growth of Lactobacillus plantarum WCFS1 on a complex medium using a genome-scale metabolic model. *The Journal of Biological Chemistry*, *281*(52), 40041–40048.](http://paperpile.com/b/MkYOqv/v8ba2)
